# In vivo transplantation of mammalian vascular organoids onto the chick chorioallantoic membrane reveals the formation of a hierarchical vascular network

**DOI:** 10.1038/s41598-025-91826-y

**Published:** 2025-02-28

**Authors:** William J. Kowalski, Shravani Vatti, Tyler Sakamoto, Wenling Li, Sarah Rose Odutola, Chengyu Liu, Guibin Chen, Manfred Boehm, Yoh-suke Mukouyama

**Affiliations:** 1 Laboratory of Stem Cell and Neuro-Vascular Biology, Cell and Developmental Biology Center, National Heart, Lung, and Blood Institute, National Institutes of Health, Bethesda, MD USA; 2 Transgenic Core Facility, National Heart, Lung, and Blood Institute, National Institutes of Health, Bethesda, MD USA; 3 Laboratory of Cardiovascular Regenerative Medicine, Translational Vascular Medicine Branch, National Heart, Lung, and Blood Institute, National Institutes of Health, Bethesda, MD USA; 4 Weill Cornell Graduate School of Medical Sciences, New York, NY USA; 5 Harvard College, Cambridge, MA USA

**Keywords:** Organoid, Chorioallantoic membrane, Vascular remodeling, Mouse embryonic stem cells, Cardiovascular models, Angiogenesis

## Abstract

The dynamic remodeling of the nascent vascular network into a mature hierarchy is essential for embryo survival. Cell behaviors and signaling mechanisms are often investigated with animal models and perfused microchannels, giving insights into this process. To support these studies and enrich our understanding, we demonstrate a complementary approach using vascular organoids. Organoids initially form a primitive endothelial plexus lined with NG2^+^/PDGFRβ^+^ mural cell progenitors containing immature pericytes, but there is no formation of large-diameter vessels covered with αSMA^+^ cells containing immature vascular smooth muscle cells (vSMCs). After transplantation to the chick chorioallantoic membrane, the network reorganizes into a branched architecture with large-diameter vessels covered by αSMA^+^ cells. We additionally show that blood flow from the host circulation perfuses the organoid. Compared with the developing skin vasculature in mouse embryos, organoids successfully recapitulate vascular morphogenesis, both in vitro and after transplantation. The model described here presents a further approach to enhance the study of vascular remodeling.

**Supplementary Information:**

The online version contains supplementary material available at 10.1038/s41598-025-91826-y.

## Introduction

Vascular branching and morphogenesis is a critical aspect of embryogenesis, yet it develops pathophysiologies that contribute to numerous disease states. Nascent capillary plexi first emerge as endothelial cell (EC) precursors migrate and coalesce to form a network of functional vessels^1^. As the vessels extend, ECs secrete platelet-derived growth factor BB (PDGFBB) to recruit pericytes, generating a honeycomb-shaped capillary network covered with perivascular cells^2^. Once formed, the primitive vascular bed undergoes intensive remodeling into a branched architecture with large and small caliber vessels arranged in a complex hierarchy of arteries, veins, and capillaries. This angiogenic remodeling is dependent on blood flow, which exposes ECs to constant forces including pressure, circumferential stretch, and wall shear stress^3–5^. Hemodynamic forces are sensed by mechanotransduction complexes and activate cell-signaling cascades, which control lumen patency^6^, directional migration of ECs^6,7^, arteriovenous specification^8,9^, and recruitment of vascular smooth muscle cells (vSMCs) to large diameter vessels^10^. However, the cellular and molecular mechanisms that drive angiogenic remodeling still need to be explored further.

Flow-dependent remodeling is supported by early investigations in which redistributed blood perfusion modified vessel caliber and mural properties^11–14^. Vessels exposed to low or no flow were likely to regress while increased blood flow enlarged lumen diameter. Surgical interventions in chick embryos provided greater evidence, where ligated or depleted vessel segments generated defects in cardiac development^15–17^, intraembryonic vasculature^18,19^, and remodeling of vitelline vessels^20^. Alterations in blood flow preceded these anomalies, demonstrating the hemodynamic influence in cardiovascular development^21,22^. In the mouse embryo, reduced blood flow or hematocrit (viscosity) led to disrupted yolk sac remodeling, establishing the significance of hemodynamic forces^10,23^. In further work with mouse embryos, deletions of cardiac genes such as *Mlc2a*^24^, *Ncx1*^25,26^, and *Nkx2.5*^27^ led to abnormal vascular structure, despite no expression in ECs. Proper blood flow, therefore, is essential to vascular remodeling and, if disrupted, can cause impaired or absent development. In vitro angiogenic research has incorporated blood flow into perfused 3D microchannel models^28^ while in vivo approaches typically apply global or cell-specific gene mutations to animal embryos^1,29,30^. Microchannel networks take advantage of instrumentation and imaging access to investigate microvascular remodeling and disease^31–33^. While studies with whole embryos provide a better representation of natural development by encompassing contributions of diverse cell types, organ functions, and environmental factors, intravital imaging of vascular remodeling in animal embryos presents challenges due to lengthy nature of these processes.

In the present study, we sought to demonstrate a combined approach that generates an in vitro vascular network and transplants it to a live embryo to undergo remodeling. We first derived vascular organoids from mouse embryonic stem cells (mESCs) and then engrafted onto the chick chorioallantoic membrane (CAM), where the 3D vessel network connected to the accessible cardiovascular system and embryo. This system maintains advantages described above, while addressing several challenges such as in vitro diminished formation of large caliber vessels and recruitment of vSMCs and in vivo difficulties accessing mammalian embryos (i.e. mice). Although zebrafish present an alternative embryo, hierarchical remodeling is limited and vascular development can progress differently from mammals^1^. Additionally, development of animal models requires significant time commitment and are subject to unintended mutation targets^34–36^. In using the accessible CAM and maintaining the organoid without modifying the chick embryo, our protocol can fill the space between the in vitro and in vivo approaches and provide a platform to support or test their results. Compared with the mouse embryonic limb skin, our model formed a vascular plexus in vitro and remodeled to a branched hierarchy with large caliber vessels and mural cells connected to the chick CAM in vivo.

## Results

### Vascular organoids derived from mouse ES cells

**Fig. 1:**
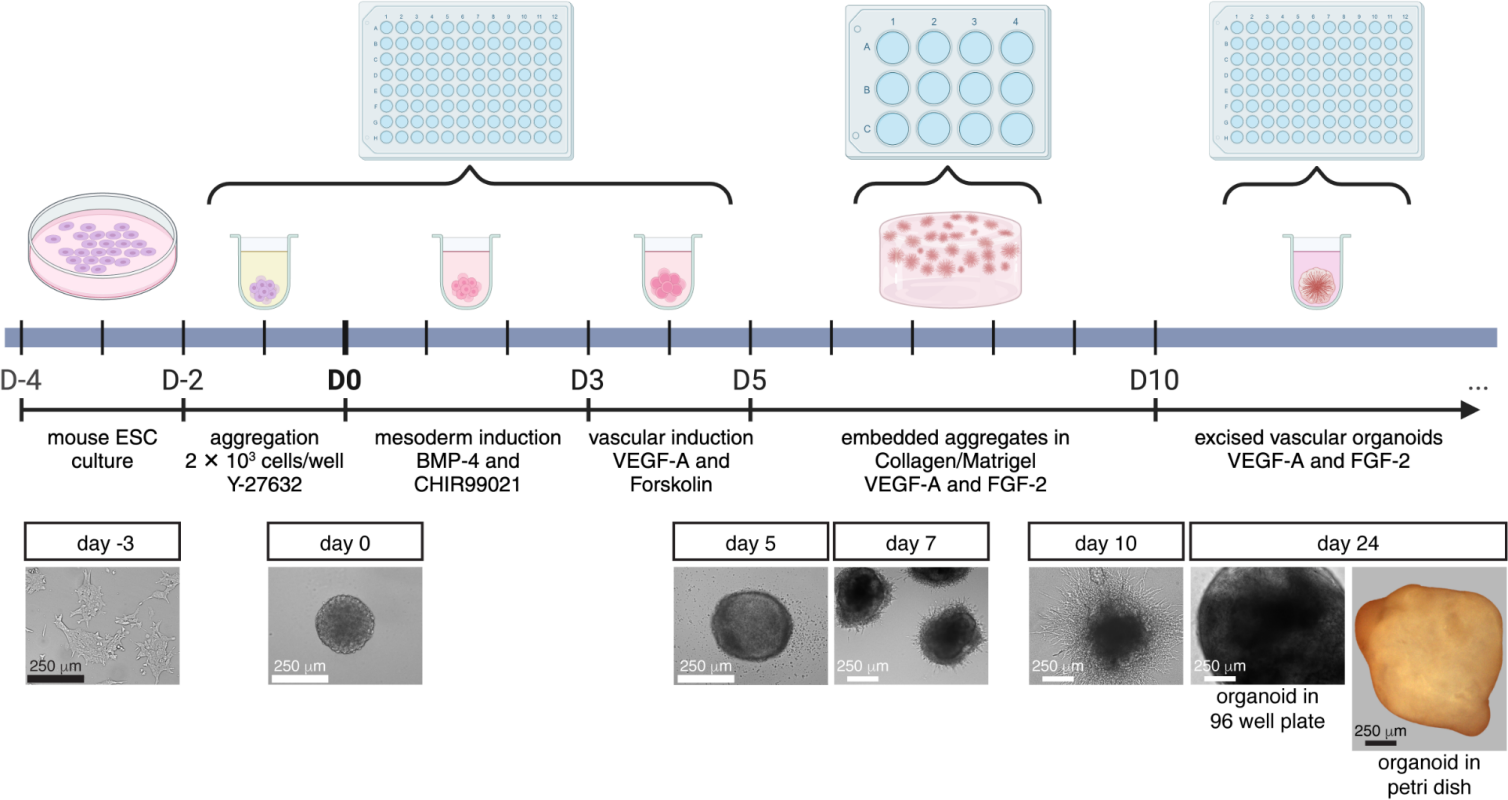
Protocol to generate vascular organoids. Mouse ESCs are cultured for 2 days and then processed through steps of aggregation, mesoderm and vascular lineage induction, embedding in hydrogel, and excised for individual organoid culture. Steps are depicted in the timeline schematic and images at different timepoints are shown below. Aggregates are formed from 2 × 10^3^ cells in ultra low attachment 96 well round bottom plates for 2 days. Day 0 (D0) is marked as the end of aggregation and start of mesoderm induction. Mesoderm and vascular lineage differentiation are performed in the same 96 well plate. Media changes are done carefully to avoid contacting the aggregate. At D5, 30–40 aggregates are embedded per hydrogel within a 12-well plate. At D10, individual organoids are excised from the hydrogel and cultured in 96 well plates. An organoid at 24 days is shown in the bottom row, in the 96 well plate and free floating in a culture dish. Diagram created with Biorender.com.

We generated vascular organoids in vitro from mESCs. Vascular organoids consisted of a 3D plexus of PECAM-1^+^ ECs with closely associated NG2^+^ cells encapsulated in an extracellular matrix of collagen I and Matrigel. Organoids were spherical and approximately 1 mm in diameter. Cultured mESCs proceeded through steps of aggregation, mesoderm induction, vascular lineage induction, embedding in hydrogel, and excising to individual organoids, requiring a total of 12 days (Fig. 1). Organoids continued in vitro culture for up to 3 weeks after segregation (31 days overall), although longer survival times are possible. Figure 1 shows an organoid after 24 days in vitro.

**Fig. 2:**
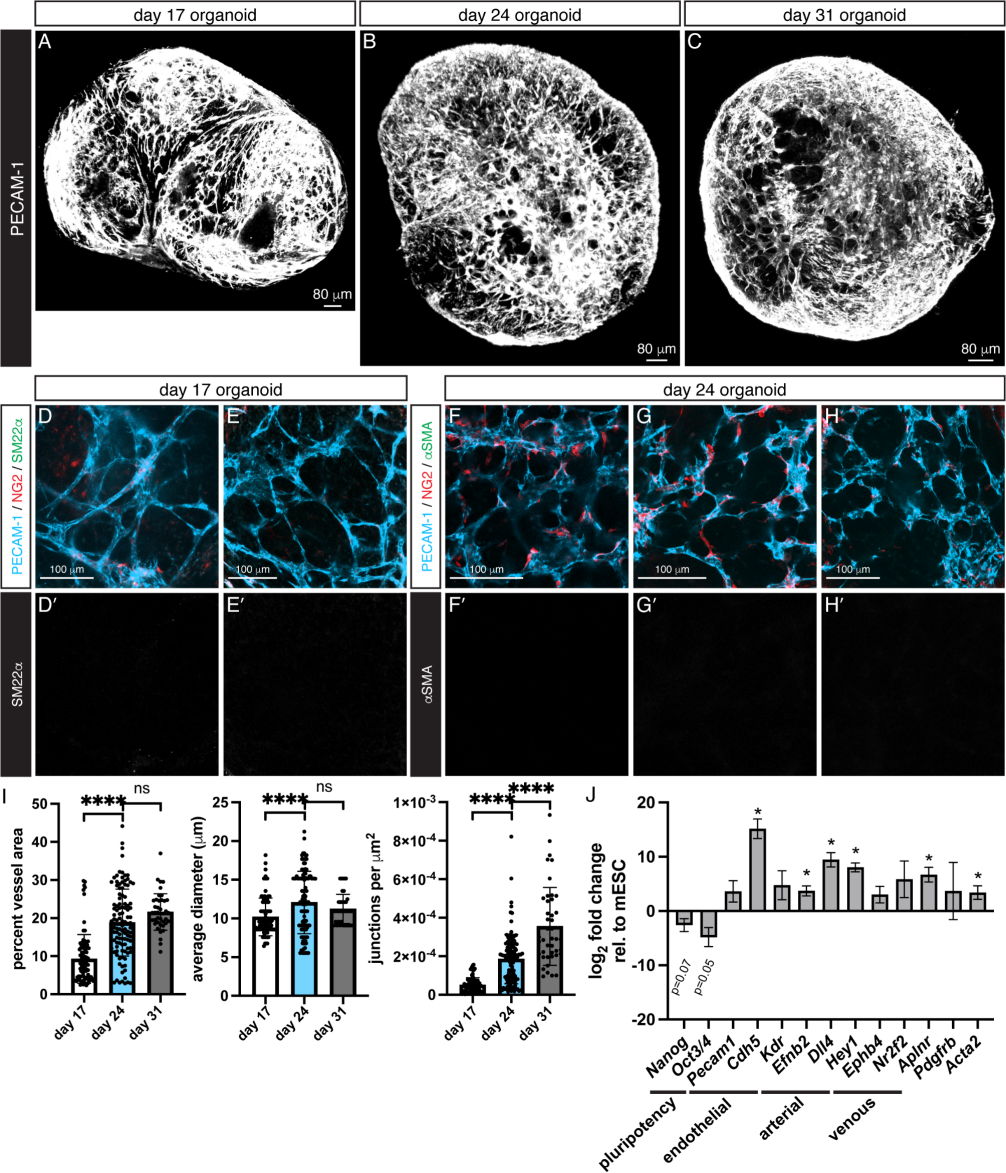
In vitro vascular organoids. Images of organoids depicted at day 17, 24, and 31. (**A**-**C**) Organoids developed an extensive plexus of PECAM-1^+^ ECs. Vessel caliber and topology appeared capillary-like and did not remodel into a branched hierarchy with increased culture time. (**D**-**E**) Higher magnification of a day 17 organoid shows a network of endothelial vessels with closely associated NG2^+^ mural cell progenitors. (**D’**-**E’**) No SM22α^+^ cells enveloped the organoid vessels. (**F**-**H**) Images of a day 24 organoid show that the vessel networks underwent sprouting angiogenesis, but architecturally remained a capillary plexus. (**F’**-**H’**) αSMA^+^ cells did not develop along these vessels. (**I**) Organoid vessel networks expanded and increased complexity between day 17 and day 24, but did not undergo remodeling from day 24 to day 31. Data are mean ± SD, unpaired t-tests are shown, ****p < 0.0001. Multiple images were analyzed per organoid and each dot represents one image, *N* = 6 organoids and *n* = 91 images at day 17, *N*/*n* = 11/113 at day 24, and *N*/*n* = 5/43 at day 31. (**J**) Fold change in gene expression of day 21 organoids relative to mESCs. Data are log_2_(fold change) ± SE. Expression of pluripotency genes decreased, *p*-values are displayed. Vascular genes increased expression in organoids, consistent with development of vessel networks. Unpaired t-tests are shown, *p < 0.05, *n* = 2–4 experiments.

The formation of mESC aggregates marked day 0 of organoid culture (Fig. 1). We applied ROCK inhibitor to promote aggregation and self-renewal of ESCs^37,38^. After 2 days, our aggregates were 250–350 µm in diameter. These aggregates were notably larger than those developed from human induced pluripotent stem cells (hiPSCs)^39^, however they formed reproducible organoids with PECAM-1^+^ ECs and NG2^+^/PDGFRβ^+^ mural cell progenitors such as immature pericytes (Fig. 2D-E’, NG2 as a pericyte marker; Figure S2, PDGFRβ as a pericyte marker). Furthermore, in our attempts to reduce the number of cells or aggregation time, aggregates tended to have rough edges or break apart. The present 2 × 10^3^ starting cell number and 2 day aggregation time proved the most reliable for generating organoids. Trials to produce multiple aggregates in a single well of a 6 or 12 well low attachment plate resulted in aggregate fusion, even with frequent gentle trituration of cells. Therefore, we formed single aggregates in separate wells of 96 well ultra low attachment round bottom plates. Media changes were performed carefully to avoid touching or disturbing the aggregates.

Following aggregation, mESCs were induced to mesodermal fate with BMP-4 and Wnt signaling and then to vascular lineage through VEGF-A exposure and Forskolin treatment (Fig. 1). During these steps, aggregates increased in size slightly and became denser. After induction (on day 5), aggregates were embedded in hydrogels to form vascular networks. We embedded 30–40 aggregates per hydrogel. VEGF-A and FGF-2 were applied to promote network growth and formation of ECs and mural cell progenitors. After 2 days, vessel sprouts emanated from the perimeter of aggregates (Fig. 1). By day 10, vessel networks had formed and made multiple interconnections between structures across the hydrogel (Fig. 1). Excised networks were cultured as individual vascular organoids. About 7 days post-isolation, organoids were fully encapsulated and appeared as 1 mm diameter spheres. We did not observe changes in organoid size as culture progressed past day 17 (Figs. 1 and 2).

### In vitro organoids form a vascular plexus

To assess vascular cell types and network architecture, we stained organoids with PECAM-1 (ECs), NG2 (pericytes), and αSMA (vSMCs). Although we use NG2 and αSMA to identify pericytes and vSMCs, respectively, these cells are2 likely a mixture of immature mural cells and pericytes. Their prominent mural cell qualities are further demonstrated by the direct association with EC networks. We therefore refer to these cells as “mural cell progenitors” or by their antibody expression throughout this study. Dense, 3D endothelial vessel networks formed within organoids and expressed PECAM-1 (Fig. ). Vessels extended throughout the organoid and formed a plexus similar to the nascent vasculature of E13.5 limb skin (Figure S1). NG2^+^ cells were associated with the endothelial networks, localized to branches and junction points (Fig. 2), an arrangement that closely resembles the E13.5 limb skin vasculature. This pattern of ECs and mural cell progenitors containing immature pericytes suggest that the in vitro organoid follows an organization program similar to embryonic vascular development. While we did not observe organoid growth after day 17, the vessel area, number of network junctions, and vessel diameters increased by day 24, indicating plexus expansion (Fig. 2I). Although the vessel junctions increased between day 24 and day 31, we did not find further changes to vessel architecture or formation of a branched hierarchy. The day 24 organoid network resembles the E13.5 limb skin vasculature. As with the limb skin vasculature, we did not find αSMA^+^ cells along these vessels (Fig. 2).

In day 21 organoids, we analyzed mRNA expression of pluripotency and vascular lineage genes (Fig. 2J). We found that pluripotency markers *Nanog* and *Oct3/4* were reduced relative to mESCs, though the fold change did not meet significance threshold (Fig. 2J). Pan-endothelial gene *Cdh5* increased expression, indicating EC formation in our organoids. Arterial transcripts *Efnb2*, *Dll4*, and *Hey1* were all upregulated, as was venous marker *Aplnr*. Expression of vSMC gene *Acta2* was increased, suggesting some vSMC differentiation, although this was undetectable by immunohistochemistry.

### Organoid-CAM model: vascular organoids were transplanted and connected to the chicken chorioallantoic membrane (CAM)

**Fig. 3:**
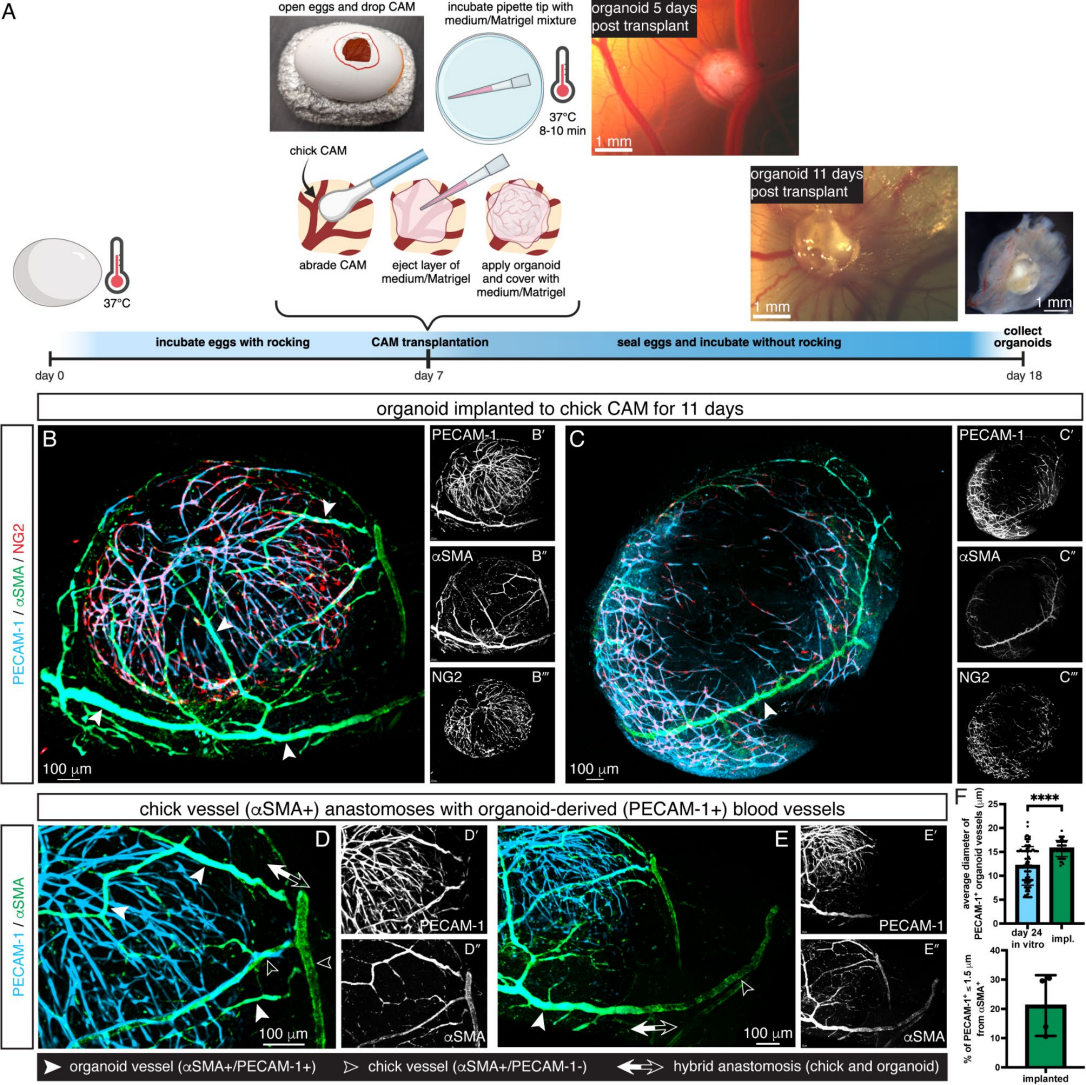
Vascular organoids transplanted to chick CAM. (**A**) Schematic protocol of CAM transplantation. Eggs were incubated to day 7, the CAM was dropped, and the shell was windowed for access. Organoids were implanted onto the CAM with a pre-incubated matrix. Transplants were reincubated for 11 days. Created with Biorender.com. (**B**-**D**) Organoids were harvested, stained, and imaged to analyze changes to vascular morphology. Organoids showed angiogenic remodeling and patterning after CAM implantation. Vessels displayed a branched hierarchy with a greater number of associated NG2^+^ cells, indicating mural cell progenitors or immature pericytes. Large diameter vessels wrapped with αSMA^+^ cells formed within the organoid (solid arrowheads). The endothelial layer of these vessels is organoid-derived (PECAM-1^+^), while the αSMA^+^ source cannot be determined from staining. (**B’**-**B’’’** and **C’**-**C’’’**) Individual markers further show mural cell covered organoid vessels. (**D**-**E**) Anastomoses between the chick host and organoid implant were observed. Double positive vessels arose from organoid-derived cells (solid arrowheads) while αSMA^+^ vessels with no underlying PECAM-1 staining are chick derived (open arrowheads), as PECAM-1 does not stain chick ECs. Double arrowheads identify anastomoses, where αSMA^+^ cells continuously envelop both chick (PECAM^−^) and organoid (PECAM^+^) vessels. (**D’**, **D’’**, **E’**, **E’’**) Single staining images depict these anastomoses. (**F**) Analysis of organoid vasculature after implantation showed larger average diameters compared with in vitro and close association with αSMA^+^ mural cells. Data are mean ± SD, *N* = 4 organoids, *n* = 49 images for average diameter analysis, unpaired t-test, ****p < 0.0001.

Previous studies have shown that the human iPSC-derived vascular organoids form hierarchical vascular networks, including large-diameter vessels with vSMC coverage, when transplanted into the kidney capsules of immunodeficient *NOD/SCID/IL2Rγ*^*null *^(NSG) mice for 2–3 months^40^. Here, we transplanted mESC-derived vascular organoids into the chicken chorioallantoic membrane (CAM) system, resulting in rapid formation of hierarchical vascular networks (Fig. 3A). A window was opened on the egg shell (day 7) to allow the transplantation of one mESC-derived vascular organoid (Fig. 3A). After 11 days of incubation, the organoids were harvested and stained using PECAM-1, NG2, and αSMA. PECAM-1 and NG2 label the mouse-derived cells and distinguish between the organoid and chick host vessels. However, αSMA labels both mouse and chick, and therefore we cannot be certain of the origin of αSMA^+^ cells. The organoid vasculature underwent significant remodeling during CAM implantation (Fig. 3B-F). The plexus of the in vitro organoid developed into a hierarchy with more associated NG2^+^ cells throughout the endothelial network. The organoid-derived vasculature exhibited significantly increased average diameter compared to in vitro organoids (Fig. 3F), indicating the emergence of large caliber vessels. Furthermore, 21 ± 10.4% of PECAM-1^+^ areas were localized with αSMA^+^ areas, demonstrating close association between mural cells and organoid ECs consistent with vascular wall remodeling (Fig. 3F). Importantly, we found the appearance of αSMA^+^ cell-covered large-diameter vessels within the engrafted organoid (Fig. 3B-C). These vessels were hybrid, made of both the organoid and chick host. We found continuous αSMA^+^ vessels with both unlabeled (chick-derived) and PECAM-1^+^ (organoid-derived) intima (Fig. 3B, D, E). These anastomoses, as well as the remodeling, suggest a functional connection between the organoid and chick host.

**Fig. 4:**
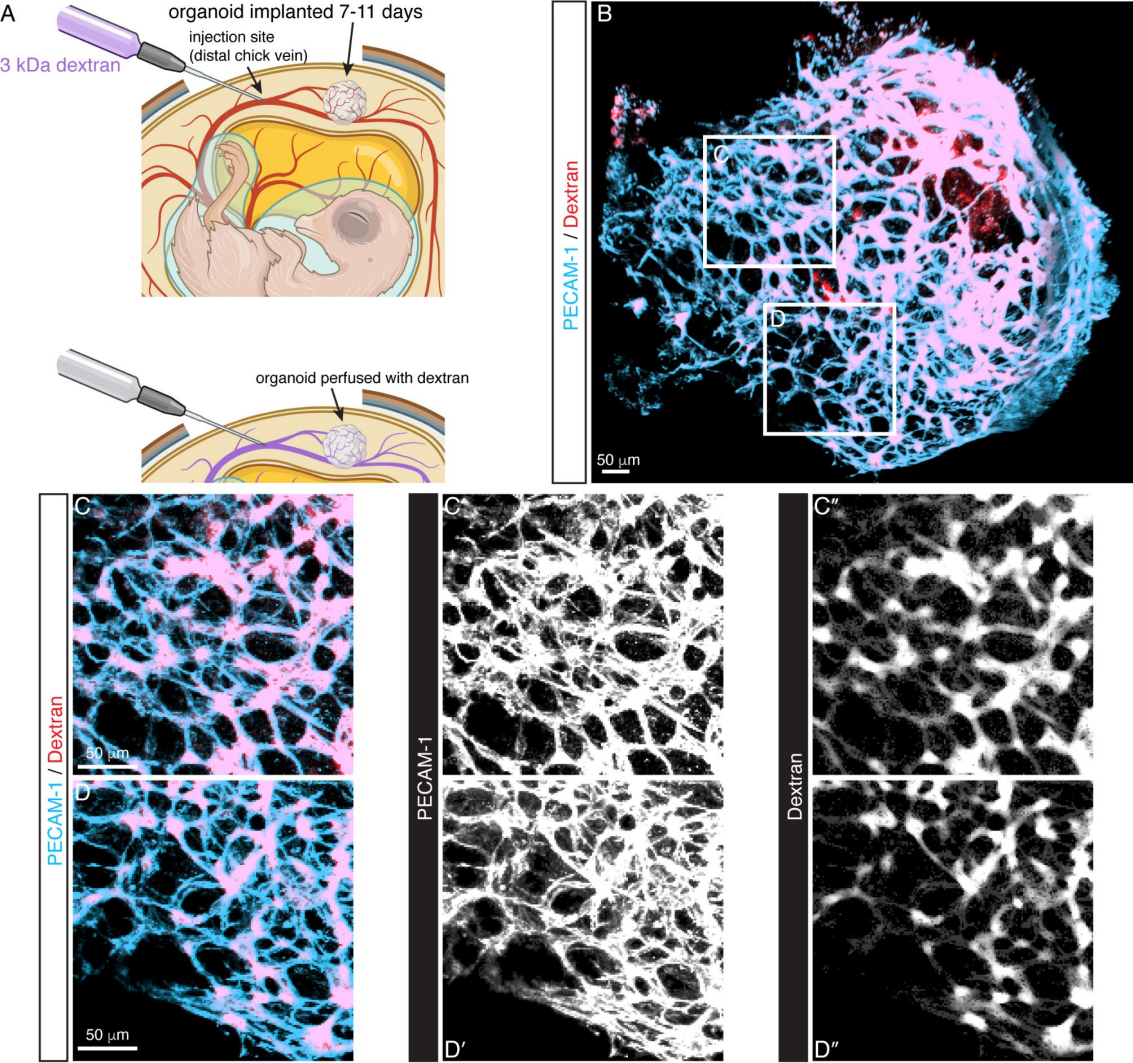
Dextran injections show blood flow connections between the chick host and organoid. (**A**) Sketch of the injection protocol. After CAM implantation for 11 days, a chick vein was injected with 3 kDa fluorescent dextran. Created with Biorender.com. (**B**) Image of PECAM-1 labeled organoid perfused with dextran after injection. Dextran is present within the endothelial network, indicating a blood flow connection between the chick embryo host and engrafted organoid. (**C**-**C’’** and **D**-**D’’**) Higher magnification of boxed areas in (**B**) depicts dextran sequestered within the organoid endothelial network.

We further examined a blood flow connection between the organoid vessels and chick vessels by dextran injection. We injected fluorescently labeled dextran into a chick vein and then harvested the organoid for imaging (Fig. 4A). Dextran was located within the organoid vasculature, indicating flow between the host and graft. Dextran is localized to the PECAM-1^+^ staining, suggesting a flow connection rather than leaking into the surrounding CAM (Fig. 4B-D). These injections support that the anastomoses observed in Fig. 3 are true functional connections between the host and organoid. During CAM implantation, the organoid vasculature developed a topology resembling the E15.5 mouse limb skin vasculature (Figure S1). Similar angiogenic programs that guide remodeling from the immature plexus (E13.5) to the branched hierarchy architecture (E15.5), likely occur within the organoid during CAM implantation. Blood flow from the chick host cardiovascular system is vital to this process.

## Discussion

Vascular organoids formed and maintained a capillary plexus in vitro, topologically resembling the immature network of the E13.5 limb skin (Figure S1). Mural cell progenitors containing immature pericytes lined blood vessels and expressed markers NG2 and PDGFRβ (Fig. 2, Figure S2). These may represent potential bleb-shaped pericytes, given their location at branch points and along capillaries and their mesh-like, thin-strand appearance^41,42^. The role of pericytes in forming primitive blood vessels is yet unclear, but they are indispensable to remodeling^2^. We did not observe other mural cell types in vitro, where αSMA and SM22α staining was undetectable. At day 21, in vitro organoids had reduced expression of pluripotency markers and upregulated pan-endothelial *Cdh5*, relative to undifferentiated mESCs (Fig. 2). Arterial genes increased significantly, and this differentiation may be driven by the VEGF-A treatment. However, venous gene *Aplnr *also increased expression, indicating both arterial and venous ECs are present. EC differentiation from stem cells in 3D produces a more heterogenous EC mix, and generating pure populations would require specific protocols^43^. Pericyte gene *Pdgfrb* was expressed, although the fold change error was broad. Despite undetectable αSMA staining, the gene *Acta2 *was upregulated, suggesting some cell transcription. These disparities between transcript levels and protein expression could relate to the stage of cell development, where organoid mural cells comprise an assortment of progenitors, immature pericytes, and immature vSMCs. In the case of αSMA, expression occurs in multiple cell types, including vSMCs, myofibroblasts, pericytes^2,42^, and mesenchymal cells^44,45^. Additional investigation of mural cells within the organoid is required to determine their identity and course of maturation.

Remodeling of the organoid plexus to a branched vessel hierarchy only occurred after transplantation to the chick CAM, supporting blood flow as the primary factor regulating the formation of hierarchical vascular networks. Dextran injections to the chick vitelline vessels perfused into the organoid vasculature, confirming functional connection between the host embryo and graft (Fig. 4). Organoid ECs were positive for PECAM-1 while chick ECs had no marker, allowing easy identification of organoid-derived vessels. After engraftment, organoid vessels reshaped to longer segments and larger diameters. NG2^+^ cells maintained association with ECs. Significantly, αSMA^+^ cells wrapped large diameter, organoid-derived vessels. We cannot determine, however, the source of αSMA^+^ cells, if it is organoid or chick, as our αSMA antibody does not differentiate. Future experiments can apply fluorescently labeled mESCs to determine its origins. The remodeled organoid vasculature displays similar hierarchy to the E15.5 limb skin vasculature, where large diameter, vSMC wrapped vessels branch toward smaller arteries and eventually capillaries (Figure S1). Our combined approach of in vitro vascular organoid culture followed by in vivo CAM engraftment successfully recapitulates the vasculogenic and angiogenic remodeling events in embryonic vascular development.

Organoids are collections of multiple cell types in 3D culture derived from stem cells and self-organized to resemble the structure and carry out functions of the represented organ^46^. Developed for a variety of organ systems, organoids are typically generated in vitro by applying specific growth factors, timing, extracellular matrix (ECM), and other cues^47–49^. Blood vessel organoids have been developed from hiPSCs and applied to study vessel development^50^ and diabetic pathology^40^. Our organoids originate from mESCs, though we could retain several procedures from the initial hiPSC protocol, including growth factors, media, and ECM embedding^39^. Treatment with VEGF-A is key for blood vessel formation^1^ while exposing stem cells to serum-containing media and FGF-2 specifies pericyte and vSMC lineages^51,52^. To adapt for mESCs, we modified aggregation methods and used a low attachment 96 well plate to avoid merging of aggregates. In a study on vessel development, hiPSC-derived organoids were engrafted to the chick CAM, similar to the approach described here^50^. These organoids were generated using fewer growth factors and did not require ECM embedding, simplifying the protocol. Vasculogenesis occurred within a ring along the periphery of the organoid, with a large inner mass of mesenchymal cells. Our organoids, however, developed a more expansive vessel network in vitro (Fig. 2). Vascular sprouting and network formation may be promoted by ECM embedding in our protocol. We attempted organoid generation with individual hydrogel droplets^49^, but found that embedding multiple aggregates in larger hydrogels produced better organoids. After engrafting to the chick CAM, our organoids developed large and small caliber vessels in a branched hierarchy. In the previous study, transplantation to the CAM did result in some wall maturation including vSMC coverage, but a hierarchy was not seen^50^. The enhanced remodeling we observed may relate to the greater vessel plexus formed in vitro, our transplantation protocol, which does not use a nylon mesh, or the time spent attached to the CAM. Importantly, we compared our vascular organoid platform to vessel development in the embryonic limb skin and found it recaptured the vasculogenic and angiogenic phases.

We elected the chick CAM as our host system for its accessibility, ease of use, and short-term implantation^53^. Additionally, the chick embryo is naturally immunocompromised; T and B lymphocytes and monocytes do not mature until E18^54^. In previous organoid-CAM transplantations, vasculature within the organoid was either absent or host-derived^55–58^. These studies applied kidney, cardiac, or brain organoids. In a tumor organoid model that included some ECs derived from mesodermal progenitor cells, connections with the CAM vessels were observed, but retained a capillary-like appearance^59^. A different host site, the mouse kidney capsule, is frequently used for organoid transplant. While these models demonstrate organoid maturation and infiltration of host vessels, they require extended implantation periods and immunocompromised mice^60,61^. Due to the immunocompromised host, organoids can form teratomas, prematurely ending experiments. Additional approaches for establishing flow within organoids have been demonstrated in vitro with EC-lined microchannels and 3D printing^62,63^.

The organoid-CAM model described here forms the vessel plexus and organizes into a branched hierarchy similar to the in vivo mammalian embryo. Organoids differentiate NG2^+^ mural cell progenitors in vitro and develop αSMA^+^ covered vessels once engrafted. Capturing this process with intravital microscopy would further our understanding of cell migrations and the transition from capillaries to arterioles and venules. Studies with mammalian embryos are limited to fixed tissues at single timepoints, given their *in utero *development. These single snapshots can breed confusion or even errors when considering dynamic events such as vascular remodeling. Vertebrate species such as zebrafish and chick, however, are amenable to live imaging. With chick, current ex ovo culture methods maintain embryos for up to 15 days and can be combined with confocal or light sheet microscopy to visualize remodeling in real time^64^. After implantation onto the CAM, organoids receive blood flow from the chick embryo host, which is essential for remodeling. Using surgical manipulations to redistribute flow can provide more support for flow-dependency and quantify hemodynamic forces^65^. Applying these techniques to the present model would provide valuable data to supplement our current understanding from mouse embryos. In a further objective, developing gene-edited mESCs for deriving and implanting organoids provides a system to investigate vascular genes essential for remodeling. Genes such as *Foxo1*, *Erg*, *Eng*, *Ephrin-B2*^66–69^ are expressed in cells other than ECs and vascular cells, and the phenotype etiology is unclear. While EC-specific Cre models can overcome some of these issues, drivers present in non-vascular ECs (e.g. endocardial cells in the heart) confound results. Deriving a vascular organoid from gene-edited mESCs sequesters the mutation to organoid cells, maintaining a normal host genotype and blood flow. Vascular phenotypes can thus be attributed to organoid cells, establishing expression within ECs and vascular cells as the prominent factor. While initial experiments can interrogate genes previously demonstrated to produce vascular phenotypes in mouse embryos, the generation of multiple organoids and short-term CAM implantation present the opportunity to screen for new genetic targets. In using mouse-derived organoids, as opposed to human stem cells, a more direct comparison with mouse embryo data can be achieved. Applying our organoid-CAM protocol to visualize remodeling and understand the genetic contribution of vascular cells will provide supporting data to current mammalian models and enhance our understanding of vascular biology.

Several refinements should be considered to enhance our model’s relevance to other research applications or address limitations. In this study, we applied antibody staining to characterize cell types within our organoid. However, discriminating between pericytes and vSMCs solely by immunostaining and morphology is challenging. These cell types potentially share a common origin, have overlapping differentiation pathways, and express similar biomarkers^52,70^. Particularly at the transition from arteriole to capillary, pericytes express αSMA^41^. While we anticipate that the NG2^+^/PDGRFβ^+^ cells in our organoid are pericytes and αSMA^+^cells after implantation are vSMCs, these cells are more accurately classified as “mural cell progenitors”. Further study is required to identify their roles as mural cells and terminal differentiation^71^. During remodeling after engraftment, organoid vessels enlarge and acquire αSMA^+^ cell covering. While PECAM-1 only labels the mouse-derived EC layer, αSMA labels both mouse and chick cells, obscuring their origin. Determining the source of these cells, whether migrated from the surrounding chick vasculature or differentiating from the organoid, is an intriguing question. Chick-derived αSMA^+^cells would exhibit how neighboring cells are recruited and migrate, while cells arising from the organoid could address differentiation of mural cells and the potential generation of vSMCs. Currently in our lab, we are exploring fluorescent reporter mESCs or using mESCs engineered with a vSMC tag^72^ to delineate organoid vs. chick sources and resolve this issue. Our current in vitro organoid generation and CAM implantation are both carried out under normoxia. Mammalian embryo development, however, occurs under lower oxygen tension, due to *in utero* development, circulatory shunts, and the mixing of embryonic and placental blood. Controlling ambient oxygen could modify organoid development and better mimic the physiologic environment. In a previous study combining mesodermal progenitors with tumor cells, lowering the oxygen tension produced a more uniform distribution of mesoderm-derived capillary-like structures, suggesting further investigation^59^. Additionally, given the essential inputs of blood flow to vascular remodeling, hemodynamics within the organoid, as well as chick and mouse embryos, should be explored with particle image velocimetry^73,74^ or computational fluid dynamics^75,76^. Finally, the organoid and utility of our model may be improved by protocol modifications such as rocking culture during in vitro development and substituting a synthetic scaffold for Matrigel during embedding and implantation. The composition of Matrigel is undefined and its biochemical and mechanical properties vary^77^. Replacing this component with a defined hydrogel could improve organoid performance and maintain consistency. Of note, we attempted CAM implantations with fibrin glue, but found that this method formed a barrier to vascular connections and ultimately led to organoid demise. Performing experiments to examine these issues can strengthen our organoid-CAM platform and make it adaptable to other research aims.

## Methods

### Mouse embryonic stem cells

Cryopreserved V6.5 mESCs^78^ were thawed and cultured without feeders as described previously^79^. One vial of 1 × 10^6^ mESCs were plated in a 0.1% gelatin coated 60 mm dish. Cells were cultured for 2 days and grew to about 80% confluency. ESC medium: Knockout DMEM (Gibco 10829018), 15% ESC qualified FBS (Gibco 16141079), 1X nonessential amino acids (Gibco 11140050), 1X Penicillin-Streptomycin (Gibco 15140122), 1X GlutaMAX (Gibco 35050061), 10 mM HEPES (Gibco 15630080), 0.1 mM β-mercaptoethanol (Gibco 21985023), 1000 U/ml LIF (Millipore Sigma ESG1107).

### Aggregation

MESCs were washed with 0.5 mM EDTA (Invitrogen 15575020) in PBS (Gibco 14190250) and collected with 1 ml Accutase (Gibco A1110501). Six (6) ml aggregation medium was added and viable cells were counted using Trypan blue staining (Invitrogen T10282). MESCs were diluted to 20 × 10^3^ cells/ml with aggregation media plus 5 µM Y-27632 ROCK inhibitor (Tocris 1254). MESCs were added at 100 µl/well (2 × 10^3^ cells per well) to 96 well ultra-low attachment round bottom plates (Sigma CLS7007) to form aggregates. Aggregates were incubated at 37 °C and 5% CO_2_ for 2 days. Aggregates were 250–350 μm in diameter and formed smooth edges (Fig. 1). Aggregation medium: KnockOut DMEM/F-12 (Gibco 12660012), 20% KnockOut serum replacement (Gibco 10828028), 1X nonessential amino acids, 1X Penicillin-Streptomycin, 1X GlutaMAX, 0.1 mM β-mercaptoethanol.

### Mesodermal and vascular lineage induction

After 2 days of aggregation, the medium was aspirated from each well with a P200 pipette. To induce mesoderm, 100 µl of N2B27 medium plus 30 ng/ml BMP-4 (Peprotech 120-05ET) and 12 µM CHIR99021 (Tocris Bioscience 4423) was added. Cells were incubated for 3 days. The medium was aspirated and replaced with 100 µl N2B27 plus 100 ng/ml VEGF-A (Peprotech 100 − 20) and 2 µM Forskolin (Sigma F3917). This medium was changed the next day. N2B27 medium: 50% DMEM: F12 (Gibco 10565018), 50% Neurobasal Medium (Gibco 21103049), 1X Penicillin-Streptomycin, 1X GlutaMAX, 1X B27 supplement minus vitamin A (Gibco 12587010), 1X N2 Supplement (Gibco 17502048).

### Embedding aggregates

Aggregates were embedded in Collagen/Matrigel hydrogels within 12 well flat bottom tissue culture treated plates (Falcon 353043). The number of wells required was calculated such that each well holds 30–40 aggregates. The hydrogel was composed of a media solution, PureCol (Advanced Biomatrix 5005), and growth factor reduced Matrigel (Corning 356231) in a 1:2:1 media: PureCol: Matrigel ratio. The media solution was prepared from 10X DMEM (Sigma D2429), Ham’s F-12 (Gibco 11765054), GlutaMAX (Gibco 35050061), 1 M HEPES (Gibco 15630080), 0.1 N NaOH (Advanced Biomatrix 5078), and 7.5% Sodium Bicarbonate (Gibco 25080094), in a 10.10:24.65:1.00:2.03:13.45:1.58 mixture ratio, respectively. PureCol followed by Matrigel were added to form the hydrogel solution. The media, PureCol, Matrigel and hydrogel solution were all kept on ice during the procedure. A 500 µl layer of hydrogel was added to each required well of the 12 well plate and incubated 37 °C for 2 h or until it solidified. Each layer was therefore 125 µl media solution, 250 µl PureCol, and 125 µl Matrigel. After solidification, aggregates were transferred to a conical tube with a P1000 pipette. Aggregates settled by gravitation at room temperature for 10 min. The medium was aspirated with a P1000 and then P200 pipette and aggregates were resuspended in hydrogel solution. The volume of hydrogel solution was calculated such that each layer contained 30–40 aggregates and there was an integer number of 500 µl layers. For example, 1500 ml hydrogel solution added to 100 aggregates generates 3 layers. One layer (500 µl) of aggregates + hydrogel was added directly on top of each solidified hydrogel layer in the 12 well plate. Aggregates were uniformly distributed by rocking the plate. The plate was incubated 37 °C for 2 h or until the new layer solidified. After solidification, warmed 1 ml Stempro medium + 15% FBS (Gibco 10270106), 100 ng/ml VEGF-A, and 100 ng/ml FGF-2 (Peprotech 100-18B) was added per well. The embedded aggregates were incubated for 5 days and medium was changed every other day. After 2 days, aggregates began to sprout and after 5 days, extensive branching was displayed (Fig. 1). Stempro medium: StemPro-34 SFM (Gibco 10639011), 1X Stempro-34 nutrient supplement, 1X Penicillin-Streptomycin.

### Organoid culture

After 5 days embedded in hydrogels, individual organoids were excised and placed in a 96 well ultra-low attachment round bottom plate for further culture. To excise, the medium was aspirated from the 12 well plate using a P1000 pipette and a single hydrogel was loosened by gently scraping around its circumference using a P10 pipette. The plate was inverted and the loosened hydrogel dispensed onto the lid of a 10 cm dish. Medium was returned to the remaining hydrogels and the plate was re-incubated during excision. The recovered hydrogel was placed under a stereomicroscope and individual organoids were dissected using two sterile 30 gauge needles and moved to the 96 well plate using a P1000 pipette with a cut end to enlarge the bore size. Stempro medium + 15% FBS, 100 ng/ml VEGF-A, and 100 ng/ml FGF-2 (100 µl per well) was added to each individual organoid. Organoids were cultured at 37 °C and 5% CO_2_. Medium was changed every other day.

### Immunohistochemistry

Organoids were washed in cold PBS and fixed in 4% PFA/PBS for 1 h at room temperature. After washing in PBS, organoids were immersed in blocking buffer (10% heat inactivated goat serum + 0.2% TX100) for 2 h at room temperature and then incubated with primary antibodies diluted in fresh blocking buffer overnight at 4 °C. The following day, organoids were washed with 2% heat inactivated goat serum + 0.2% TX100 and then incubated with secondary antibodies diluted in blocking buffer for 1 h at 4 °C and protected from light. Organoids were washed with 2% heat inactivated goat serum + 0.2% TX100 followed by PBS at room temperature. Tissue clearing was performed with serial incubations in 20%, 40%, 60%, and 80% glycerol for 2 h each at room temperature. Organoids were then incubated in clearing solution (54% benzyl alcohol and 46% glycerol^80^) at room temperature for 2 h, transferred to fresh clearing solution, and stored until imaging. All incubations were performed on a rotating mixer. Fluorescent microscopy was carried out on a Leica TCS SP5 confocal. Organoids were placed in a glass bottom dish filled with clearing solution for imaging. Primary and secondary antibodies are listed in Table S1.

Organoid vascular networks were analyzed with Angiotool^81^. We acquired one z-stack per organoid with a 10X/0.4 NA objective. To generate images for analysis, we calculated the maximum intensity projection for each group of five z slices. Multiple images per organoid were therefore processed, and each dot in Fig. 2I represents one image. A total of *N* = 6 day 17, *N* = 11 day 24 organoids, and *N* = 5 day 31 organoids were analyzed. Engrafted organoids were processed in the same fashion with Angiotool to measure average diameters of the PECAM-1^+^ vessels only. We did not measure the % vessel area and junction density, as we could not reliably delineate the organoid boundary from the CAM in our images. To assess mural cell coverage, we first generated binary masks of the αSMA and PECAM-1 channels (Oxford Instruments Imaris 9.9.0). We then applied 3D distance transforms to identify the total volume of PECAM-1^+^/αSMA^+^regions separated by less than 1.5 μm^82^. This value was normalized to the total PECAM-1^+^ volume to quantify the fraction of organoid-derived vessels associated with mural cells. We included a small distance offset to account for PECAM-1 and αSMA that may not strictly coincide. The 1.5 μm dimension is equivalent to one pixel width in our images. *N* = 4 organoids were analyzed. Statistical analysis using unpaired t-tests were performed, *p* < 0.05 was considered significant.

### RT-qPCR

Total RNA was extracted from the organoids as previously described^34^. First-strand cDNAs were synthesized with SuperScriptIII reverse transcriptase (Invitrogen) using random hexamer primers according to the manufacturer’s instruction. The same starting mass of RNA was used for all experiments. Quantitative mRNA expression analysis was performed with LightCycler 96 (Roche) using FastStart Universal SYBR Green master (Roche). A 10 µl reaction was performed, with cDNA diluted 1:4 and a final primer concentration of 250 nM. Reactions were run in triplicates and a Ct difference greater than 0.5 cycles was rejected. Gene expression was analyzed following the 2^− ΔΔCt^ method, first normalized to housekeeper *Hprt1 *and then to undifferentiated mESCs^83^. Fold changes were compared with unpaired t-tests, *p* < 0.05 was considered significant. The PCR primer sequences are listed in Table S2.

### CAM transplantation and dextran injection

We transplanted day 21–31 vascular organoids onto the day 7 chick CAM and maintained them for 11 days. Fertile white Leghorn eggs (University of Connecticut Department of Animal Science) were incubated in a horizontal orientation with rotation at 37 °C and 50–60% humidity for 7 days (HovaBator 1588 with automatic egg turner). For implantation on day 7, we mixed one volume of Matrigel (Corning 356230) with two volumes of Stempro medium + 15% FBS, 100 ng/ml VEGF-A, and 100 ng/ml FGF-2 on ice. We drew up 100 µl of the Matrigel/medium mixture into P200 pipette tips (one per planned implantation), ejected the filled tips into a 10 cm dish, and incubated for 8–10 min at 37 °C to prepolymerize the ECM (Fig. 3). To prepare the eggs and access the CAM, we followed a method similar to Palmer et al. part 1^84^. After gaining access to the CAM, we lightly abraded the area for transplant with a cotton tipped applicator and then ejected 60–80 µl of the prepolymerized Matrigel/medium mixture onto abraded site. Using an embryo spoon, we lifted a vascular organoid from the 96 well plate, placed it onto the freshly prepared site, and then covered with the remaining 40 –20 µl ECM mixture (Fig. 3). The egg window was sealed with tape and egg returned to the incubator without rotation. After 10 min, we unsealed the egg and placed a drop of HBSS + 1X Penicillin-Streptomycin onto the organoid. Eggs were then re-sealed and incubated for 11 days. For immunohistochemistry, we microdissected the organoid with a small area of attached chick CAM and washed in PBS prior to fixation.

To assess connections between the host and organoid vasculature, we injected 3 kDa dextran-Texas red (Invitrogen D3328) into the chick embryo. Eleven (11) days post-transplantation, eggs were unsealed and the shell window was enlarged. A chick embryo vitelline vein distal to the organoid was selected for injection. Microneedles pulled from glass capillary pipettes (Narishige PC-10) were connected via silastic tubing to a 20 gauge needle and 1 ml syringe (Becton Dickinson). Microneedles were mounted to a micromanipulator and injections were performed under a stereomicroscope. One-two minutes after injection, the needle was extracted and organoid/CAM was excised with microscissors. The excised organoid/CAM was washed with PBS before fixation and immunohistochemistry.

## Electronic supplementary material

Below is the link to the electronic supplementary material.

Supplementary Material 1

## Data Availability

The datasets generated during and/or analyzed during the current study are available from the corresponding author on reasonable request.
