# Supplementary material for: In vivo transplantation of mammalian vascular organoids onto the chick chorioallantoic membrane reveals the formation of a hierarchical vascular network
Source: Sci Rep. 2025 Feb 28;15:7150. doi: 10.1038/s41598-025-91826-y (PMC11871353; doi:10.1038/s41598-025-91826-y)
Supplement: Supplementary file 1 — Supplementary Material 1 [file 41598_2025_91826_MOESM1_ESM.pdf]

Supplementary Information

In vivo transplantation of mammalian vascular organoids onto the chick chorioallantoic membrane reveals the formation of a hierarchical vascular network

William J. Kowalski[1], Shravani Vatti[1, 4], Tyler Sakamoto[1, 5], Wenling Li[1], Sarah Rose Odutola[1, 5], Chengyu Liu[2], Guibin Chen[3], Manfred Boehm[3], Yoh-suke Mukouyama[1*]

[1]Laboratory of Stem Cell and Neuro-Vascular Biology, Cell and Development Biology Center,

[2]Transgenic Core Facility,

[3]Laboboatory of Cardiovascular Regenerative Medicine, Translational Vascular Medicine Branch,

National Heart, Lung, and Blood Institute,

National Institutes of Health, 10 Center Drive, Bethesda, MD, USA

[4] Weill Cornell Graduate School of Medical Sciences, New York, NY, USA

[5]Harvard College, Cambridge, MA, USA

[*]Author for correspondence:
E-mail: mukoyamay@mail.nih.gov
Tel: (301) 451-1663

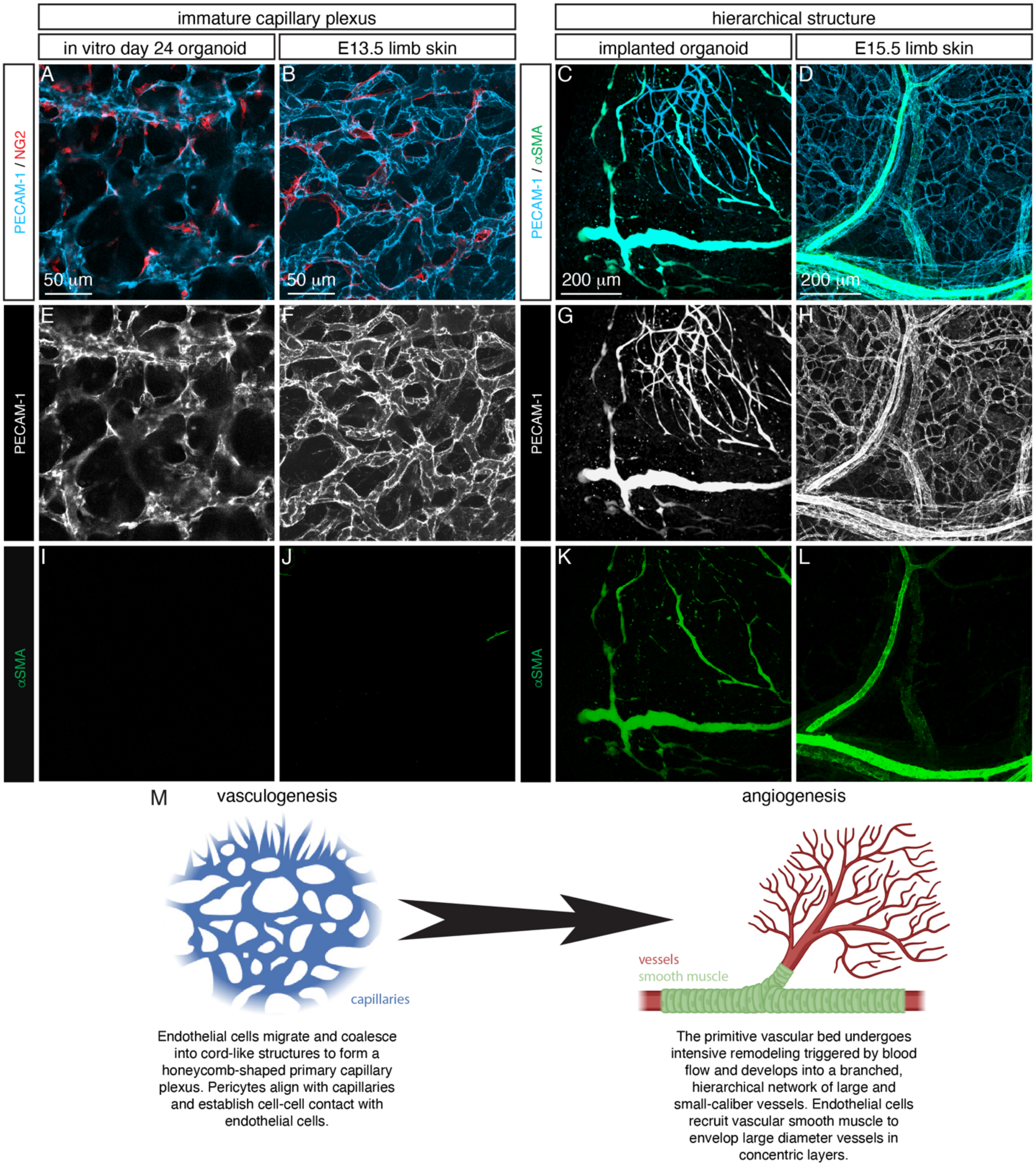

Figure S1. The immature capillary plexus of in vitro organoids, once implanted to the chick CAM, underwent architectural remodeling similar to the mouse embryonic limb skin. A and B) The vessel network of the organoid comprised a honeycomb structure and cell distribution congruent with the E13.5 limb skin. $NG2^{+}$ mural cell progenitors associated with endothelial branches and junctions in both systems. C and D) After implantation to the chick CAM, the organoid vasculature remodeled to a branched hierarchy, forming a topology comparable to the E15.5 limb skin. Large diameter

vessels enveloped with αSMA^+ cells are present in both models. E-H) Single-channel endothelial staining further shows hierarchical changes between the organoid (E and G) and limb skin (F and H). I-L) αSMA^+ mural cells were absent or minimal in the immature vasculature (I and J), but were recruited in later stages (K and L). M) The illustration depicts the transition from a vessel plexus formed by vasculogenesis to a mature branched geometry formed through angiogenic remodeling. Created with Biorender.com.

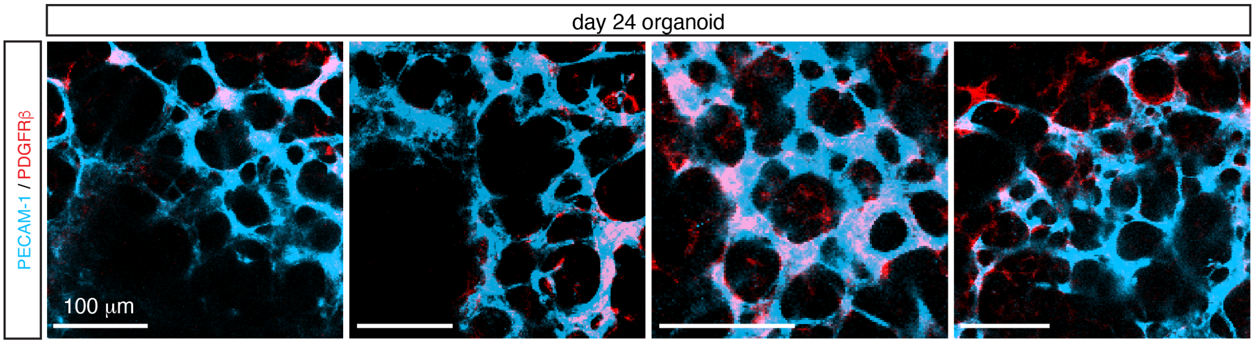

Figure S2. Mural cell progenitors associated with endothelial networks expressed PDGFRβ in addition to NG2 (see Figure 2).

Table S1. Primary and secondary antibodies for immunostaining

antibody	host	vendor/product	dilution
αSMA-FITC	Mouse, clone 1A4	Sigma, F3777	1:500
NG2	Guinea Pig	Wm. Stallcup, Sanford Burnham Prebys	1:300
NG2	Rabbit	Millipore, AB5320	1:300
PECAM-1	Armenian Hamster, clone 2H8	Millipore, MAB1398Z	1:300
PDGFRβ	Rabbit	Wm. Stallcup, Sanford Burnham Prebys	1:300
SM22α	Rabbit	Abcam, ab14106	1:500

secondary antibody	fluorophore	vendor/product	dilution
Goat anti-Armenian Hamster	Cy3	Jackson Immuno, 127-165-160	1:250
Goat anti-Guinea Pig	AF488	Jackson Immuno, 106-546-003	1:250
Goat anti-Rabbit	AF647	Jackson Immuno, 111-605-144	1:250
		Invitrogen, A21244	1:250

Table S2. Gene-specific oligonucleotide primers for qRT-PCR [1,2]

gene	sense (5' to 3')	antisense (3' to 5')
Nanog	TCTTCCTGGTCCCCACAGTTT	GCAAGAATAGTTCTCGGGATGAA
Oct3/4	CACCATCTGTCGCTTCGAGG	AGGGTCTCCGATTTGCATATCT
Pecam1	AACAGAAACCCGTGGAGATG	GTCTCTGTGGCTCTCGTTCC
Cdh5	TCAACGCATCTGTGCCAGAGAT	CACGATTTGGTACAAGACAGTG
Kdr	TTTGGCAAATACAACCCTTCAGA	GCAGAAGATACTGTCACCACC
Efnb2	AGGAATCACGGTCCAACAAG	GTCTCCTGCGGTACTTGAGC
Dll4	TTCCAGGCAACCTTCTCCGA	ACTGCCGCTATTCTTGTCCC
Hey1	GCGCGGACGAGAATGGAAA	TCAGGTGATCCACAGTCATCTG
EphB4	GGTCAGCGCTCTGGACAAGATG	AGCCGAATCCAGCCGCTGCAA
Nr2f2	TGGAGAAGCTCAAGGCACTG	ACGAAGCAAGAGCTTTCCGA
Aplnr	TGGTGTTCCGTTCCACAGAC	GGTCACTACAAGCACCACGA
Pdgfrb	TTCCAGGAGTGATACCAGCTT	AGGGGGCGTGATGACTAGG
Acta2	GTCCCAGACATCAGGGAGTAA	TCGGATACTTCAGCGTCAGGA

References

1 Kinugasa-Katayama, Y. *et al.* Tmem100-BAC-EGFP mice to selectively mark and purify embryonic endothelial cells of large caliber arteries in mid-gestational vascular formation. *Genesis* **59**, e23416 (2021). https://doi.org:10.1002/dvg.23416

2 Poh, Y. C. *et al.* Generation of organized germ layers from a single mouse embryonic stem cell. *Nat Commun* **5**, 4000 (2014). https://doi.org:10.1038/ncomms5000
